# Wideband Current Transducer Traceable Calibration up to 10 A and 1 MHz

**DOI:** 10.3390/s24082608

**Published:** 2024-04-19

**Authors:** Mohamed Ouameur, Daniela Istrate, François Ziade

**Affiliations:** Laboratoire National de Métrologie et d’Essais, 78197 Trappes, France; daniela.istrate@lne.fr (D.I.);

**Keywords:** current measurement, current transducer, calibration, pulse current, wideband transducer, Pearson current transformer, calculable current shunt, uncertainty

## Abstract

Energy efficiency is an important issue in industry, especially with the ever-increasing consumption of electrical energy. The power quality and the traceability of metering devices are essential when integrating energy metering systems for energy efficiency. This management requires an understanding of electrical current events such as pulse and transient currents. Current transducers are widely used to measure these electrical current events up to a few megahertz. Their use makes it possible to measure not only the main current flowing through the transducer, but also the bypass current that affects electrical equipment. Calibration of these sensors up to a few megahertz then becomes an essential step. Currently, most calibration methods are limited to 100 kHz frequency for a current of 10 A. This paper presents an improvement of a traceable calibration methodology for current transducers up to 10 A and 1 MHz, thus increasing, by 10 times, the current level for such high frequency applications. This calibration methodology is based on a metrological traceability chain (uninterrupted link to the International System of Units) with respect to a calculable current shunt and is currently the only traceable method for calibrating current transducers at 10 A and up to 1 MHz. The uncertainty obtained for the transimpedance ratio is less than 0.2%, which is considerably reduced with respect to the existing capabilities.

## 1. Introduction

Electrical current abnormal events (pulsed and transient) always affect the voltage and current in AC and DC circuits. The stable-state values of these events are usually reached after the event period. In electrical networks, various sources such as switching operations in circuit breakers [1,2], lightning strikes [3,4] or electromagnetic pulses [5] can generate short-duration high-amplitude accidental events and therefore affect the quality of electrical power. During these events, voltage and current increase several times compared to nominal operating values. Under these conditions, it is difficult for electrical equipment to withstand voltage spikes and overcurrents, as they are typically designed for permanent voltage and current levels. The disturbances are also associated with abnormal frequency content that can be as high as several kilohertz [4,5]. Equipment characterization for these disturbance modes is therefore important for industry and device manufacturers, and tests are required by international standards such as the IEC 60060 family for voltage [6], and IEC 62475 for current [7].

Moreover, the technological innovations in semiconductors (e.g., SiC and GaN) and converter architectures allow reaching high switching frequencies (beyond 100 kHz) and increased levels of nominal voltage and current (hundreds of volts and amperes, respectively) with high efficiency, to ensure a bidirectional flow of energy. This allows the emergence of new applications such as renewable energies and electric transportation (e.g., electric vehicles, tram, metro or train) with significant impact on the power quality. New issues related to disturbances generated by the AC/DC or DC/AC high power convertors appeared (e.g., short-circuits, transient occurrences and ripples) by increasing the risk to the safe operation of grids and by stressing equipment (mainly in DC grids where there is no more zero crossing voltage as in AC networks). The need to ensure traceable and accurate measurements on wideband distorted signals exists and the on-going research projects [8,9] prove it. 

Wideband current transducers (such as inductive or zero-flux sensors) are extensively used as references because of their measurement capabilities: large current ranges (up to a few tens of kiloamperes) for a short duration (µs, so bandwidth up to few megahertz), their galvanic isolation, and their ability to measure low and high currents with the same coil size [10,11]. The wideband current transducers are generally designed for high value currents (kA) and short durations (MHz frequency band). Current transducers provide either current or voltage output proportional to the flowing current. This paper focus on the latter. 

The appropriate calibration of such transducers with the best uncertainties requires a high level of current in a wideband frequency. The increased current level allows obtaining increased output voltages, which reduce the influence of the measurement noise. Calibrations must be performed at frequencies as high as possible to cover the major part of transient event spectra.

Today, the calibration of wideband current transducers is limited either in amplitude or in frequency. Standard sources delivering a few tens of kiloamperes in the form of pulses or transients do not exist, while the generation of permanent high currents (tens of amperes) at frequencies above 100 kHz is still difficult and rare, and the characterized references up to 1 MHz and amplitudes above 1 A are not available.

This paper focuses on the transducers generating a voltage as the output signal proportional to the current flowing through it. Therefore, the transimpedance ratio, *K* expressed in V/A, can be defined as the ratio between the RMS voltage *U_T_* measured at the transducer terminals and the RMS current *I_T_* flowing through it, such that:

(1) $$K=\frac{U_{T}}{I_{T}}$$

The intensity of an unknown electric current can be obtained by measuring the output voltage of the transducer using calibrated voltmeters and by knowing the transimpedance ratio, *K*. 

The calibration of a current transducer used for pulsed and transient events at high current levels (a few kiloamperes) and frequencies (a few tens or hundreds of megahertz) is not possible. Therefore, this calibration is based on two pillars:

− The determination of its transimpedance ratio in terms of magnitude for the whole frequency operating range;
− The metrological current transducers, whose properties remain constant in time (stability, drift, etc.) and have a linear response to current level with acceptable mechanical dimensions for high currents [12,13].

In recent years, several methods have been developed to calibrate wideband current transducers. The first type is based on the use of a sinusoidal reference current with a frequency covering the interval from 50 Hz to 1 MHz and amplitudes up to 1 A. For amplitudes higher than 1 A (up to 10 A) the calibration frequency of the sinusoidal current is limited to 100 kHz [14,15,16,17]. These methods rely generally on the use of resistive standards. For currents up to 10 A, the resistive standards (current shunts) are generally calibrated up to 100 kHz. For frequencies above 100 kHz (typically up to 1 MHz), these resistive standards are limited in current amplitude to 1 A. These limitations are due to the generation of 10 A currents up to 1 MHz and calibration of standards, mainly 10 A current shunts up to 1 MHz.

An example of this type of method is given in [15]. The French National Metrology Laboratory (LNE) team explains the traceable method implemented to calibrate wideband current transducers up to 1 A, 1 MHz based on a direct comparison. The reference for current measurement was a 1 Ω resistor metrologically characterized at fixed frequencies of 100 kHz and 1 MHz for a nominal current of 1 A. The reference used, method and current level make the measurements very sensitive to the noise and to the electromagnetic interferences. Additionally, the current transducers to be calibrated usually have low transimpedance ratios (which can be less than 1 mV/A) worsening the noise influence. The expanded relative measurement uncertainties of the transimpedance ratio are ±1.3% for 1 A, 100 kHz and ±2.0% for 1 A, 1 MHz. One solution, which is included in the developments presented in this paper, is the increase of the current level. 

The second type of calibration method is based on the generation of high standard currents (a few kiloamperes) at 50 Hz or 60 Hz frequency [18,19,20,21,22,23,24,25] that applies only to certain types of inductive transducers like Rogowski coils. When the first type of calibration method is applied, the transimpedance ratio is obtained at a low current level, which considerably increases the measurement uncertainty. When the second type of calibration is applied, the transimpedance ratio is obtained at operating currents near to the nominal values. However, the calibration of transducers at 50 Hz or 60 Hz frequency remains insufficient to characterize their response to abnormal current events.

This paper describes the improvement of the calibration method based on a direct comparison with a standard current shunt for wideband current transducers. The approach relies on the determination of the transimpedance ratio of the transducer at AC currents up to 10 A intensity and a wide frequency range (up to 1 MHz) by comparison with a resistive reference. Existing calibration limitations are overcome by generating a current of 10 A up to 1 MHz using a commercial amplifier associated with an impedance adapter, and the development of a method of calibrating current shunts to 10 A and 1 MHz by direct comparison with a standard calculable current shunt. A significant reduction in uncertainties has been achieved thanks to the standards used, the coefficient calculation method and the particular care taken with the assembly (synchronized acquisition of voltages, short cables in the electrical circuit and reduction of stray capacitances). Consequently, this paper presents mainly a method that is the only one capable of traceably calibrating wideband current transducers at 10 A and 1 MHz with a relative uncertainty of less than 0.2%. Secondarily, the paper also presents a calibration method for 10 A current shunts for frequencies up to 1 MHz (previously limited to 100 kHz) with an expanded uncertainty on the impedance modulus variation of less than 78 µΩ/Ω up to 1 MHz.

## 2. Wideband Current Measurement Setup and Methodology

**Figure 1:**
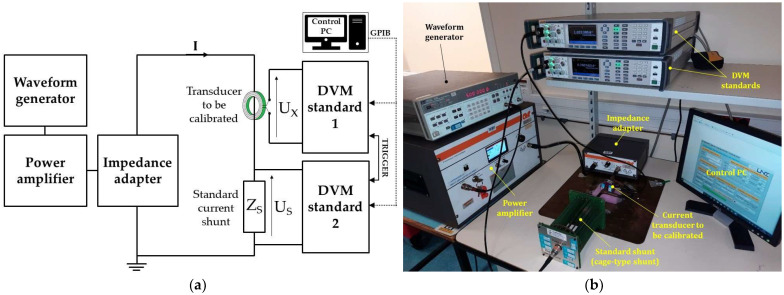
Current transducer calibration setup (**a**) scheme and (**b**) example.

The wideband transducer calibration measurement setup (Figure 1a) consists of a waveform generator, a power amplifier and associated impedance adapter, the standard shunt, two calibrated digital voltmeters (DVMs), and a control computer (PC). 

The transimpedance ratio *K* of the unknown current transducer is determined by knowing the variation of the impedance modulus of the standard resistor (*Z_S_*) and by measuring simultaneously the RMS AC voltages across the standard resistor (*U_S_*), respectively, at the output of the current transducer to be calibrated (*U_X_*). The same alternating current *I* flows through the current transducer and the standard resistor. The transimpedance ratio *K* is then given by the following equation:

(2) $$K=\frac{U_{X}}{U_{S}}\cdot \left|Z_{S}\right|$$

Despite the calibration principle based on comparison, the novelty of the method presented in this paper relies mainly in the ensured traceability up to 10 A and 1 MHz. This is achieved by using a source able to deliver the necessary current at a high frequency (Figure 1) and by using the calculable current shunt standard with a known response.

The solid-state amplifier composing the supply source ensures an instantaneous bandwidth and a high gain. Its use associated with the impedance adapter avoids mismatch impedance when the standard current shunt is connected to the setup.

The calculable current shunt is a custom-made resistor specially designed for operation at 10 A at MHz frequency range with errors as low as possible. It constitutes the primary standard [26] and it is used to calibrate cage-type shunts [27] that became the reference measurement standard [28]. Thus, the use of the latter in the calibration of wideband transducers is associated with very low uncertainties and contributes to the creation of metrological traceability. More details are given in the following section.

**Figure 2:**
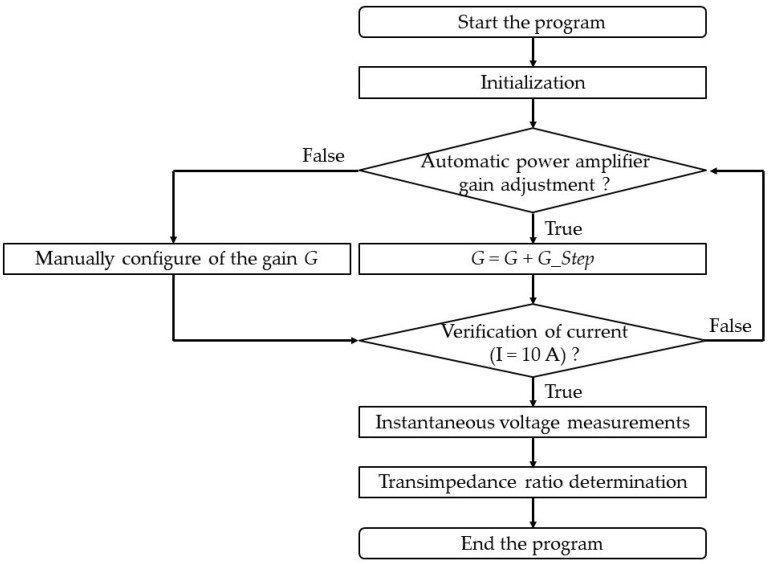
Program algorithm for the current transducer calibration. *G* is the power amplifier gain and *G_Step* is the step of the power amplifier gain (Ex, = 1%).

The methodology applied in a regular wideband current transducer calibration consists of adjusting the supply source for the desired sinusoidal current and in measuring the RMS voltages of the reference standard and the unknown transducer synchronously. The output voltages are measured by two DVMs calibrated in the interval 10 mV–10 V up to 1 MHz using thermal transducers [29,30]. A ground plane is used to ensure the same reference for all devices in the measurement circuit and to avoid ground loops. The distribution of instantaneous voltage values measured for the same current level mainly indicates the stability of the supply source. This instability does not influence the calibration of the transducers because of the comparison method; the transimpedance ratio *K* being given as a function of the voltage ratio and standard impedance modulus (Equation (2)). Consequently, the synchronization of voltage measurements becomes the critical factor. This program also allows the automatic (or manual) adjustment of the power amplifier gain to achieve the required current (10 A) by measuring the output voltage of the standard shunt. This adjustment is made carefully to avoid injecting a current higher than the nominal current of the standard shunt. The program algorithm is shown in Figure 2. The transimpedance ratio is the average of the instantaneous values (for example, 50 measurements with an acquisition time of about 1 min, since it takes about 1 s between each two successive measurements). The number of measurements is to be adjusted to reduce the standard deviation of the measurement repeatability, which is mainly related to the synchronization of the two DVMs, the stability of the standard and calibrated sensors and the measurement setup. However, the methodology presented remains valid when using another resistive standard, provided that it is calibrated at 10 A and up to 1 MHz. In this case, the corresponding uncertainties should be calculated using the uncertainty budget presented in this paper.

## 3. Traceability to the International System of Units (SI)

**Figure 3:**
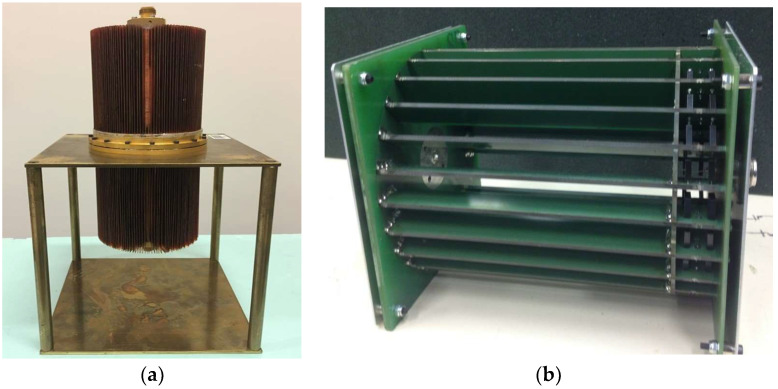
Coaxial current shunt standards. (**a**) Calculable current shunt of 10 A. (**b**) Cage-type current shunt of 10 A.

The current measurements are linked to the volt and ohm units of the SI. The DVMs are calibrated by means of AC–DC thermal converters, the primary standards for AC voltages. For voltages below 10 V, the expanded uncertainties on the voltage up to 1 MHz are less than 5·10^−5^·U (V). The cage-type current shunt used is calibrated both in DC and AC. The DC resistance value is traceable to the French national resistance standard derived from the von Klitzing constant, *R_K-_*_90_, via the quantum Hall effect [31]. The AC impedance value of the cage-type current shunt is calibrated by direct comparison with the calculable current shunt (Figure 3a). The calculable current shunt has a coaxial structure based on a resistive disk [26]. It is placed perpendicular to a coaxial line between the center conductor and the shield (outer conductor). Two type-N coaxial connectors complete the structure, the first to inject the electric current and the second to measure the voltage at the terminals of the shunt.

The resistance value of the calculable shunt is based on the resistive disk made by vacuum evaporation deposition of the resistive alloy on an insulating substrate. Two vacuum deposition techniques have been tested for the resistor design: either by “Joule effect” evaporation with heating of the substrates to a high temperature (e.g., 280 °C), or by cathode sputtering. Both processes do not guarantee the same electrical and thermal properties between the deposited thin film and the bulk alloy, but they do allow obtaining a flat and homogeneous surface, which is necessary for the calculable aspect of the shunt. The analytical model is composed of a number of coaxial sections whose geometric dimensions and material properties are known [26]. This model allows defining the variation of the impedance modulus. The real and imaginary parts of the impedance of the calculable shunt are obtained as a function of frequency using the analytical model that is detailed in [26]. The nominal resistance of the current shunt is 80.3 mΩ. At 1 MHz, the AC–DC difference and phase angle *ϕ* are less than 397 ppm and 1.44 mrad, respectively. These values depend mainly on the quality of the vacuum deposition of the resistive disk. 

The significant stabilization time of the calculable current shunt impedance (a few tens of hours) does not allow it to be used as a standard for the routine calibration of current transducers. In this paper, the calculable shunt is used as a primary standard, thus reducing its usage rate to avoid a significant drift of its analytical model.

A cage-type current shunt is then used as the transfer standard. It has a nominal value of 80 mΩ, and it is shown in Figure 3b. This geometry is distinguished by resistors mounted on fins across a PCB substrate [27]. Before being used as the transfer standard, this shunt was fully calibrated up to 1 MHz. The calibration of the cage shunt covers a frequency range from 10 kHz to 1 MHz for a nominal current of 10 A. This calibration allows determining the two main parameters: the phase angle *ϕ* and the variation of the impedance modulus at a given frequency with respect to its DC resistance value *R_DC_*. This last parameter is called the AC–DC difference and is noted as *δ*. These two parameters are calculated by:

(3) $$\left\{\begin{array}{c} \delta (f)=\frac{|Z_{Shunt}|-R_{DC}}{R_{DC}}\\ \;\\ \phi (f)=arctan\left(\frac{Im(Z_{Shunt})}{Re(Z_{Shunt})}\right) \end{array}\right.$$

where *R_DC_* is the DC resistance of the current shunt impedance *Z_Shunt_*, *δ* is the AC–DC difference of the impedance *Z_Shunt_* at the frequency *f*, *ϕ* is the phase angle of the impedance *Z_Shunt_* at the frequency *f*, and Re(*Z_Shunt_*) and Im(*Z_Shunt_*) are the real and imaginary parts of the impedance *Z_Shunt_* at the frequency *f*.

The use of the analytical model of the calculable shunt [26] to determine the shunt impedance variation as a function of frequency with its associated uncertainties makes it possible to ensure low uncertainties in the calibration of the cage-type current shunt (up to 1 MHz) compared to other calibration methods [32], which are mostly limited in frequency to 100 kHz.

The calibration methods used in metrology laboratories to characterize current shunts are limited in frequency to 100 kHz for 10 A and allow the measurement of a single parameter: either the AC–DC difference or the phase angle. These methods are based on the following principles: the direct comparison method [33,34,35,36], thermal transfer method [37], potentiometer method [38] and Vector Network Analyzer (VNA) method [39]. The VNA is an instrument that measures the frequency response of a component (passive in our case) and is mainly used for RF and high frequency (typically frequencies above 1 MHz) applications. An approach to characterize current shunts has been developed and validated in [39] up to 1 MHz based on the use of the VNA and polynomial regression. The VNA is calibrated before each measurement session using a traceable calibration kit. Table 1 compares the possible uncertainties with the different calibration methods used to characterize current shunts.

For the magnitude calibration of current transducers, it is mainly necessary to characterize the AC–DC difference of the current shunts. For currents of 10 A, the calibration methods based on the direct comparison principle [33,34,35,36] and the thermal transfer principle [37] are limited in frequency to 100 kHz, and do not allow for guaranteeing the traceability of the current transducer calibration between 100 kHz and 1 MHz. This limitation is mainly due to the standards used, calibrated up to 100 kHz, and to the supply source, which does not allow the generation of 10 A and 1 MHz. Based on the VNA instrument, it is possible to obtain the impedance variation of current shunts up to a few megahertz. During these measurements, the maximum supply current of VNA ranges from 0.5 mA to 30 mA (in a power range from −25 dBm to +10 dBm) and the VNA is mainly used to evaluate frequency variation. Nevertheless, this method is based on the assumption that the current level does not affect the frequency variation of the impedance modulus, but its DC resistance value. Therefore, the measured values on the VNA are corrected by considering the DC calibration of the current shunt [39]. However, the combined uncertainties on the transimpedance ratio of the current transducers are better than 2%, which is mainly due to the possible uncertainties on the AC–DC difference with the VNA method.

**Figure 4:**
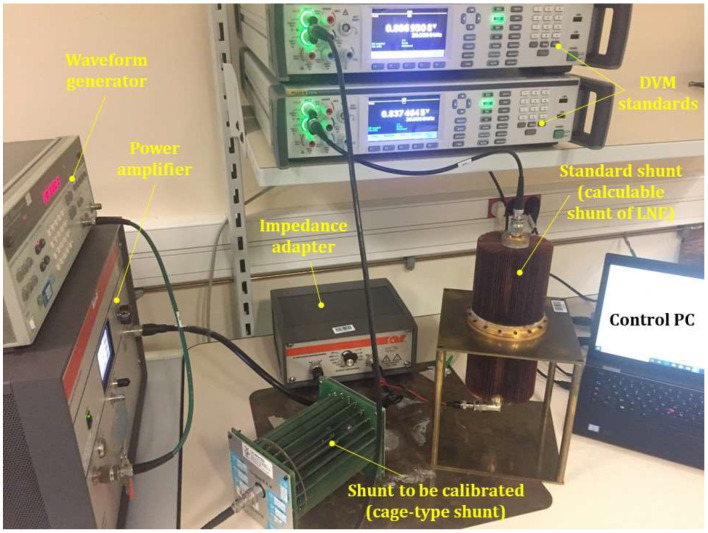
Setup of the cage-type current shunt calibration using the calculable current shunt.

Due to the limitations of existing calibration methods, the cage-type shunt was calibrated with a comparison method and the calculable current shunt as a standard [26]. The cage-type shunt was calibrated up to 1 MHz using the same principle as shown in Figure 1. Corrections were applied with respect to the temperature coefficient for the 10 A electrical current level. More details are given in the uncertainty budget calculation. Figure 4 shows the 10 A cage-type current shunt calibration.

Table 2 shows a comparison of the AC–DC difference of the calibrated cage shunt using three calibration methods: a direct comparison with the calculable shunt, a direct comparison with other standards and the VNA measurement. The chosen method to calibrate the reference measurement standard (cage-type shunt) is the direct comparison with the calculable shunt since it offers the lowest uncertainties up to 1 MHz. The expanded uncertainty related to this calibration is determined considering the uncertainty of the calculable shunt, the current generation system (waveform generator, power amplifier and associated impedance adapter), the DVMs and the repeatability. The largest uncertainty contribution is the uncertainty associated with the DVMs (78 µΩ/Ω).

## 4. Uncertainty Budget for the Calibration of Current Transducers

The uncertainty components related to the reference measurement standard (cage-type shunt), DVMs and type A components are obtained during the metrological characterization of the setup. These components are described in detail as follows:

- Standard shunt calibration uncertainty (uS_1_): This component corresponds to the cage-type shunt calibration (DC and AC) by direct comparison with the calculable current shunt (Figure 4). The expanded uncertainty in the AC–DC difference of the cage shunt impedance is 78 µΩ/Ω up to 1 MHz (Table 2). The standard uncertainty of this component is injected in the uncertainty propagation law of Equation (3) to obtain the combined uncertainty on the shunt impedance, which is less than 12 µΩ (*k* = 1) up to 1 MHz.
- Uncertainty related to the temperature effect on the standard shunt (uS_2_): Measurements are made in a controlled laboratory with a temperature variation of 23.5 °C ± 0.5 °C and humidity variation 45% ± 5%. The temperature coefficient *TC* of the cage-type shunt is 10 (µΩ/Ω)/°C. The variation of the impedance modulus due to the temperature variation is calculated by the product of the impedance value *Z*, the temperature variation around the ambient value *∆T* and the temperature coefficient *TC* [40]; its value is 1·10^−5^·Z. This correction can be neglected for the current transducer calibration. However, the uncertainty associated with this correction is considered and obtained by applying the rectangular distribution law (dividing factor is 2√3):
  (4) $$u\left({uS}_{2}\right)=\frac{∆T\cdot Z_{T0}\cdot TC}{2\sqrt{3}}=\frac{1{}^{\circ}C\cdot 80mΩ\cdot 10(µΩ/Ω)/{}^{\circ}C}{2\sqrt{3}}$$
  where *Z_T_*_0_ is the impedance modulus at the ambient temperature, *T*_0_, of the standard current shunt.
- Uncertainty related to the DVM calibration (uD_1_): The voltmeters are calibrated before measurement by checking the voltage levels (up to 10 V) as a function of frequency. The appropriate corrections are applied to the measured RMS voltage values. The expanded uncertainties are less than 5·10^−5^·U (V) up to 1 MHz.
- Uncertainty related to the DVM drift (uD_2_): The value considered for this uncertainty component is the largest and corresponds to the 200 mV range in the frequency range up to 1 MHz. Considering a rectangular distribution law, the contribution of this uncertainty component to the total budget is 6·10^−5^·U per year.
- Uncertainty associated with the DVM resolution (uD_3_): This component can be neglected because the digitizer reading is in double-precision (floating decimal with an accuracy of six digits). Considering a normal distribution law, the value of this uncertainty component is estimated to be (1 µV)/√3.
- Uncertainty related to the temperature effect on DVMs: The voltmeters are used after heating and in their temperature range. For the ambient temperature of 23.5 °C, the corrections and uncertainties related to this component are neglected (use within the temperature range specified by the manufacturer) and are not applied to each DVM.
- Uncertainty related to the crosstalk effects (uK_1_): The proximity of the transducer to the current shunt (or other transducers) might have an influence on their measurements. The uncertainty value was evaluated by increasing the distance between the transducer and the current shunt from 5 to 50 cm. The relative uncertainty of this effect is estimated to be 5.8·10^−8^·K.
- Uncertainty related to the measurement stability (uK_2_): This component is evaluated during the measurements by calculating the standard deviation of more than 50 measurements of the transimpedance ratio for transducers with nominal values of 10 mV/A and 100 mV/A. It is less than 0.11 mV/A up to 1 MHz.
- Uncertainty related to the measurement synchronization (uK_3_): The synchronization error between the two DVMs on voltage acquisitions is less than ± 50 µs. The uncertainty component is estimated by computing the standard deviation of the transimpedance ratio measured with the imposed synchronization error, and its value is 7.2 µV/A.

Table 3 summarizes the standard uncertainties (*k* = 1) used in the final uncertainty budget. Considering that the two DVMs used to measure RMS voltages *U_S_* and *U_X_* have the same uncertainties (the corrections were applied accordingly to the voltage range), the uncertainty of the transimpedance ratio is given by using the uncertainty propagation law from Equation (2):

(5) $$u^{2}\left(K\left(f\right)\right)={\left(\frac{\partial K\left(f\right)}{\partial Z_{S}\left(f\right)}\right)}^{2}\cdot u^{2}\left(uS\right)+\left[{\left(\frac{\partial K\left(f\right)}{\partial U_{S}\left(f\right)}\right)}^{2}+{\left(\frac{\partial K\left(f\right)}{\partial U_{X}\left(f\right)}\right)}^{2}\right]\cdot u^{2}\left(uD\right)+u^{2}\left(uK\right)$$

where uS, uD and uK are the overall uncertainties (*k* = 1) for the impedance of the standard shunt, the voltage measured by the standard DVM and the measuring bridge on the conversion ratio *K*(*f*).

By calculating the sensitivity coefficients, the uncertainty of the transimpedance ratio for the current transducer is then calculated:

(6) $$\begin{array}{c} u^{2}\left(K\left(f\right)\right)={\left(\frac{U_{X}\left(f\right)}{U_{S}\left(f\right)}\right)}^{2}\cdot \sum_{i=1}^{2}u^{2}\left({uS}_{i}\right)+\left[{\left(-\frac{U_{X}\left(f\right)}{U_{S}^{2}\left(f\right)}\cdot \left|Z_{S}\left(f\right)\right|\right)}^{2}+{\left(\frac{\left|Z_{S}\left(f\right)\right|}{U_{S}\left(f\right)}\right)}^{2}\right]\\ \cdot \sum_{i=1}^{3}u^{2}\left({uD}_{i}\right)+\sum_{i=1}^{3}u^{2}\left({uK}_{i}\right) \end{array}$$

where *U_X_*(*f*) and *U_S_*(*f*) are the RMS voltages measured across the current transducer and the cage-type current shunt at frequency *f*, respectively. |*Z_S_*(*f*)| is the impedance modulus of the cage-type shunt at frequency *f.* uS_i_, uD_i_ and uK_i_ are the standard uncertainties (*k* = 1) of the standard current shunt, the DVMs and the other components influencing the transimpedance ratio *K*, respectively.

## 5. Results of Wideband Transducer Calibrations

The method presented has been used to calibrate three commercially available current transducers:

- Current transducer Eurocraft B-0.1 with a nominal transimpedance ratio of 100 mV/A.
- Current transducer Pearson 101(1) with a nominal transimpedance ratio of 10 mV/A.
- Current transducer Pearson 101(2) with a nominal transimpedance ratio of 10 mV/A.

Two different current transducers, Pearson 101 (1 and 2), of the same type were calibrated to validate the reproducibility of the uncertainty results on the same type of transducers. The improved method presented in this paper and the method, by comparison with a standard shunt, existing before this paper were used. All the results are obtained with the conductor carrying the current centered with respect to the center of the current transducers. Table 4 shows the uncertainty calculation for the two inductive current transducers Eurocraft B-0.1 and Pearson 101(1). The calibration results of the current transducers Eurocraft B-0.1, Pearson 101(1) and Pearson 101(2) are presented in Table 5.

The results show very good agreement between the obtained values of the transimpedance ratio *K* given by the presented method and the results of the initial method fit in the uncertainty limits. Considering the low uncertainties of the improved method compared to the uncertainties of the method initially used for current transducer calibration, the obtained results allow validating the improvements presented in this paper.

The largest uncertainty in the global budget of the method presented in this paper is related to the stability of the measurements. For example, for the current transducer Eurocraft B-0.1, the uncertainty contributions from the standard shunt and the DVMs are less than 0.06 mV/A and 0.01 mV/A, respectively. These values are small compared to the uncertainty contribution from the measurement stability of 0.11 mV/A. Despite this large value in the uncertainty budget, this contribution has also been improved from the original method (0.4 mV/A) by using the high current level of 10 A instead of the 1 A initially used. The use of the calculable shunt (of 10 A) as a primary standard ensures low uncertainties of the resistive standard (cage-type shunt) in the whole uncertainty budget; in the first calibration method used for current transducer calibration, this uncertainty component is equal to 2 mV/A at 1 MHz. The transimpedance ratios of wideband current transducers are therefore obtained with expanded uncertainties of less than ±0.2% for 10 A and up to 1 MHz. These low uncertainties obtained with the new method allow the initial uncertainties to be reduced by a factor of 10.

## 6. Conclusions

This paper presents an improvement of the method used for the calibration of commercial current transducers up to 1 MHz. The use of the analytical model of the custom-made calculable shunt to calibrate the cage-type current shunt are crucial to reach low uncertainties of the standard up to 1 MHz. The higher current level (10 A) compared to existing methods (1 A) allows reducing the measurement noise impact and improving the measurement stability. 

The methodology applied to determine the transimpedance ratio is based on the average of more than 50 instantaneous determinations. Additionally, the synchronized acquisitions of the standard and the calibration transducers allowed us to neglect the influence of synchronization errors. The reduction of the stray capacitances by using appropriate T-connectors, a ground plane and as short as possible shielded cables contributes to lower the uncertainty. 

As a result, the transimpedance ratios of the wideband current transducers are obtained with expanded uncertainties of ±0.2% for 10 A and up to 1 MHz. Consequently, the initial uncertainties are reduced by a factor of 10 (initially ±2% at 1 A, 1 MHz). The main contribution of this paper consists of presenting a traceable method to calibrate current transducers that are high in frequency and amplitude (10 A up to 1 MHz). 

The same method presented in this paper can be used to calibrate current shunts up to 1 MHz for currents of 10 A, using the calculable current shunt as a standard. At present, there are no calibration methods to calibrate such devices up to 1 MHz for currents of 10 A; the existing methods are limited to 100 kHz. The method presented in this paper ensures uncertainties on the AC–DC difference of less than 78 µΩ/Ω up to 1 MHz.

Future improvements of this method are planned to allow the additional calibration of the phase displacement of the current transducers. The phase angle of the cage-type shunt will be linked to the analytically defined phase angle of the calculable shunt (1.44 mrad at 1 MHz).

## Figures and Tables

**Table 1:** Comparison of different calibration methods for current shunt calibration.

Calibration Method	Limit Current	Limit Frequency	Maximum Extended Uncertainty of AC–DC Difference	Maximum Extended Uncertainty of Phase Angle
Direct Comparison Method	300 mA	1 MHz	-	±200 μrad
	100 A	100 kHz	±200 μA/A (135 μA/A for 10 A) *	±50 μrad *
Thermal Transfer Method	1 A	1 MHz	±91 μA/A	-
	10 A	100 kHz	±110 μA/A	-
Potentiometer Method	20 A	200 kHz	-	±141 μrad
VNA Method	30 mA	10 MHz	±600 µΩ/Ω up to 1 MHz for shunts of 10 A	±1.6 mrad up to 1 MHz for shunts of 10 A

* The determination of the AC–DC difference and phase angle is performed by two independent methods.

**Table 2:** AC–DC difference of the calibrated cage current shunt up to 1 MHz.

Frequency (kHz)	Direct Comparison Method (with the Calculable Shunt)		Direct Comparison Method (to Another Resistance Standard)		VNA Method	
	AC–DC Difference (µΩ/Ω)	Expanded Uncertainty (µΩ/Ω)	AC–DC Difference (µΩ/Ω)	Expanded Uncertainty (µΩ/Ω)	AC–DC Difference (µΩ/Ω)	Expanded Uncertainty (µΩ/Ω)
10	0	54	1	50	4	600
20	0	67	1	60	7	600
50	1	70	20	90	18	600
100	4	72	62	135	36	600
200	11	72	-	-	72	600
500	58	73	-	-	189	600
1000	211	78	-	-	403	600

**Table 3:** Standard uncertainties (*k* = 1) used in the final uncertainty budget for current transducer calibration.

Uncertainty Component	Notation	Uncertainty Type	Maximum Estimated Uncertainty (*k* = 1)
Standard shunt calibration	uS_1_	B	12 µΩ
Temperature influence on the standard shunt	uS_2_	B	0.24 µΩ
Calibration of the DVMs	uD_1_	B	5·10^−5^·U (V)
Drift of the DVMs	uD_2_	B	6·10^−5^·U (V)
Resolution of the DVMs	uD_3_	B	0.6 µV
Crosstalk effects	uK_1_	B	5.8·10^−8^·K (mV/A)
Stability of the measurement	uK_2_	B	0.11 mV/A
Synchronization of the measurement	uK_3_	B	7.2 µV/A

**Table 4:** Combined uncertainty (*k* = 1) for the inductive current transducer magnitude calibration up to 1 MHz.

Calibrated Inductive Current Transducer	Frequency (kHz)	Sensitivity Coefficients and Standard Uncertainties Due to:										Combined Uncertainty in mV/A (*k* = 1)
		Nominal Sensitivity Coefficient of the Impedance Z	Nominal Sensitivity Coefficient of the DVMs (A^−1^)	Standard Shunt Calibration uS_1_ (µΩ)	Temperature Influence on the Standard Shunt uS_2_ (µΩ)	DVMs Calibration uD_1_ (µV)	DVMs Drift uD_2_ (µV)	DVMs Resolution uD_3_ (µV)	Crosstalk Effects uK_1_ (nV/A)	Stability of the Measurement uK_2_ (µV/A)	Synchronization of the Measurement uK_3_ (µV/A)	
Eurocraft B-0.1	10	1.25	0.16	3.61	0.24	17.50	30.00	0.58	2.90	61.37	7.22	0.06
	20	1.25	0.16	4.83	0.24	22.50	30.00	0.58	2.90	81.74	7.22	0.08
	50	1.25	0.16	5.08	0.24	22.50	30.00	0.58	2.90	91.84	7.22	0.09
	100	1.25	0.16	5.24	0.24	25.00	30.00	0.58	2.90	94.56	7.22	0.10
	200	1.25	0.16	5.25	0.24	25.00	30.00	0.58	2.90	98.47	7.22	0.10
	500	1.25	0.16	5.38	0.24	25.00	30.00	0.58	2.90	101.13	7.22	0.10
	1000	1.25	0.16	5.72	0.24	25.00	30.00	0.58	2.90	114.01	7.22	0.11
Pearson 101(1)	10	0.13	0.10	3.61	0.24	1.75	3.00	0.58	0.29	7.73	7.22	0.01
	20	0.13	0.10	4.83	0.24	2.25	3.00	0.58	0.29	8.27	7.22	0.01
	50	0.13	0.10	5.08	0.24	2.25	3.00	0.58	0.29	7.26	7.22	0.01
	100	0.13	0.10	5.24	0.24	2.50	3.00	0.58	0.29	8.31	7.22	0.01
	200	0.13	0.10	5.25	0.24	2.50	3.00	0.58	0.29	7.70	7.22	0.01
	500	0.13	0.10	5.38	0.24	2.50	3.00	0.58	0.29	7.88	7.22	0.01
	1000	0.13	0.10	5.72	0.24	2.50	3.00	0.58	0.29	8.15	7.22	0.01

Eurocraft B-0.1 and Pearson 101 are commercial inductive current transducers.

**Table 5:** Calibration results for the measured inductive current transducer up to 1 MHz.

Current Transducer	Frequency (kHz)	Transimpedance Ratio *K* (mV/A) with the Expanded Uncertainties (*k* = 2)	
		Improved Method Presented in This Paper (at 10 A)	Method Initially Used for Current Transducer Calibration (at 1 A)
Eurocraft B-0.1	10	100.90 ± 0.10	100.90 ± 1.30
	20	101.00 ± 0.20	100.90 ± 1.30
	50	100.90 ± 0.20	101.10 ± 1.30
	100	100.90 ± 0.20	100.90 ± 1.30
	200	101.00 ± 0.20	100.90 ± 2.10
	500	101.30 ± 0.20	100.50 ± 2.10
	1000	101.00 ± 0.20	101.20 ± 2.10
Pearson 101 (1)	10	10.05 ± 0.02	10.08 ± 0.13
	20	10.03 ± 0.02	10.11 ± 0.13
	50	10.11 ± 0.02	10.09 ± 0.13
	100	10.10 ± 0.02	10.24 ± 0.13
	200	10.11 ± 0.02	10.27 ± 0.21
	500	10.19 ± 0.02	10.24 ± 0.21
	1000	10.68 ± 0.02	10.63 ± 0.21
Pearson 101 (2)	10	10.08 ± 0.02	10.06 ± 0.13
	20	10.07 ± 0.02	10.06 ± 0.13
	50	10.06 ± 0.02	10.05 ± 0.13
	100	10.05 ± 0.02	10.03 ± 0.13
	200	10.08 ± 0.02	10.10 ± 0.21
	500	10.10 ± 0.02	10.06 ± 0.21
	1000	10.38 ± 0.02	10.41 ± 0.21

## Data Availability

Data are contained within the article.
